# Nature and Extent of Genetic Diversity of Dengue Viruses Determined by 454 Pyrosequencing

**DOI:** 10.1371/journal.pone.0142473

**Published:** 2015-11-13

**Authors:** Md Abu Choudhury, William B Lott, Shahera Banu, Anthony Youzhi Cheng, Yik-Ying Teo, Rick Twee-Hee Ong, John Aaskov

**Affiliations:** 1 Menzies Health Institute Queensland, Griffith University, Brisbane, Australia; 2 Institute of Health and Biomedical Innovation, Queensland University of Technology, Brisbane, Australia; 3 School of Chemistry, Physics, and Mechanical Engineering, Science and Engineering Faculty, Queensland University of Technology, Brisbane, Australia; 4 Saw Swee Hock School of Public Health, National University of Singapore, Singapore, Singapore; 5 Department of Statistics and Applied Probability, National University of Singapore, Singapore, Singapore; 6 Life Sciences Institute, National University of Singapore, Singapore, Singapore; 7 Genome Institute of Singapore, Agency for Science, Technology and Research, Singapore, Singapore; Thomas Jefferson University, UNITED STATES

## Abstract

Dengue virus (DENV) populations are characteristically highly diverse. Regular lineage extinction and replacement is an important dynamic DENV feature, and most DENV lineage turnover events are associated with increased incidence of disease. The role of genetic diversity in DENV lineage extinctions is not understood. We investigated the nature and extent of genetic diversity in the envelope (E) gene of DENV serotype 1 representing different lineages histories. A region of the DENV genome spanning the E gene was amplified and sequenced by Roche/454 pyrosequencing. The pyrosequencing results identified distinct sub-populations (haplotypes) for each DENV-1 E gene. A phylogenetic tree was constructed with the consensus DENV-1 E gene nucleotide sequences, and the sequences of each constructed haplotype showed that the haplotypes segregated with the Sanger consensus sequence of the population from which they were drawn. Haplotypes determined through pyrosequencing identified a recombinant DENV genome that could not be identified through Sanger sequencing. Nucleotide level sequence diversities of DENV-1 populations determined from SNP analysis were very low, estimated from 0.009–0.01. There were also no stop codon, frameshift or non-frameshift mutations observed in the E genes of any lineage. No significant correlations between the accumulation of deleterious mutations or increasing genetic diversity and lineage extinction were observed (*p*>0.5). Although our hypothesis that accumulation of deleterious mutations over time led to the extinction and replacement of DENV lineages was ultimately not supported by the data, our data does highlight the significant technical issues that must be resolved in the way in which population diversity is measured for DENV and other viruses. The results provide an insight into the within-population genetic structure and diversity of DENV-1 populations.

## Introduction

RNA virus populations are characterised by a high frequency of mutations introduced into their genomes; this ranges from 10^−3^ to 10^−5^ substitutions per nucleotide per round of replication [1, 2]. This effect is compounded by large population sizes and short generation times [3]. The observed high mutation rates are principally due to the error-prone nature of their virally encoded RNA-dependent RNA polymerases (RdRPs) [4]. The resulting RNA virus populations are genetically diverse, thus allowing these viruses to evolve rapidly to fill new ecological niches [5, 6]. For example, mutations in RNA genomes potentially allow RNA viruses to escape from vaccine strategies, antiviral drugs and host immune responses.

The literature suggests that increased genetic diversity facilitates virus adaptation and transmission, its dissemination in new tissues and organs, and infection in new hosts [7–10]. However, most phenotypic mutations in RNA viruses during replication are deleterious [11], and this increases the possibility of extinction during repeated population bottlenecks. RNA viruses, therefore, live on the edge of the “error catastrophe” [12], striking a delicate balance between the genetic diversity required to ensure survival in mutable and hostile host environments and the accumulation of deleterious mutations that would ultimately lead to extinction.

RNA viruses such as the dengue viruses (DENVs) show extensive intra- and inter-host genetic diversity [13]. Regular lineage extinction and replacement is one of the dynamic features of DENV [10, 14], and although most DENV lineage extinction and replacement events are associated with increased incidence of disease, the mechanisms responsible are not well understood. Recent reports of increased diversity in populations of RNA viruses associated with increased fitness [7, 9] appear to argue against most mutations being deleterious. The emergence of defective viruses into a circulating functional DENV population, including nonsense mutations in the envelope (E) gene and defective interfering (DI) viral particles, resulted in increased transmission in nature [15, 16].

DI DENV particles contain RNA genomes with large internal deletions relative to the parental virus. The residual sub-genomic fragment must be replicated in the host cell by complementation with co-infecting functional viruses. The mode of transmission of these co-infecting functional viruses causes DENV populations to frequently experience population bottlenecks. A high proportion of defective genomes within a circulating DENV population would be expected to make that population vulnerable to extinction during bottleneck events. Thus, the accumulation of defective viruses could cause DENV lineages to become extinct. To date, however, no study has determined the impact of DENVs genetic diversity on lineage turnover.

Cloning and Sanger sequencing is the gold standard technique to measure DENV intra- and inter-host genetic diversity [15, 17, 18]. However, this technique is limited by the amount of the genome that can be cloned into a single plasmid for subsequent sequencing, and by the logistics of sequencing large numbers of cloned genome fragments. Previous experiments exploring DENV intra-host genetic diversity were limited to a small set of clones (*n* = 10 to 30) containing short, amplified segments of the viral genome. Recent advances in sequencing techniques, such as next-generation sequencing (NGS), can potentially overcome these limitations [19–21] through clonally amplifying thousands of DENV template fragments without traditional cloning. NGS should more accurately identify DENVs containing short defective genomes than analogous cloning techniques. However, to our knowledge, no previously reported studies have comprehensively measured the genetic diversity of DENVs from different evolutionary histories by NGS.

The DENVs are single stranded, positive sense RNA viruses consisting of four antigenically distinct serotypes (DENV-1, 2, 3 and 4) belonging to the *Flavivirus* genus within the family *Flaviviridae*. DENV RNA genome is approximately 11Kb and contains a single long open reading frame (ORF) flanked by the two untranslated regions (5^/^ and 3^/^ UTR).The ORF encodes a polyprotein that is co- and post-translationally cleaved by host and viral proteases into three structural proteins [the capsid protein (C), a membrane associated protein (M), and an envelope protein (E)], and seven non-structural proteins (NS1, NS2A, NS2B, NS3, NS4A, NS4B and NS5) [22, 23]. The E protein contains domains I, II, and III (which form the ectodomain) and the C-terminal hydrophobic domain (C-term), which has distinct structural functionalities in the folding and assembly of the protein on the surface of the virion and potentiate different antibody responses in humans [21].

To quantify and characterise genetic variations between extinct and circulating lineages, we sequenced the E genes of 13 DENV-1 from four lineages by Roche/454 pyrosequencing. Myanmar DENV-1 isolates were used in this study because the laboratory has had access to a large collection of DENV isolates since 1998. At the time of analysis, lineages A and B had become extinct, and lineages C and D continued to circulate. Lineage A contained the first DENV-1 isolate; this became extinct in 1998—about the same time that lineages B and C appeared. No examples of lineage B have been recovered since 2002; however, lineage C was still circulating in 2008. Lineage D, first detected in 2006, was also still circulating in 2008 [24].

## Materials and Methods

### Study population

Viruses were recovered from acute phase sera from dengue patients admitted to the Yangon Children’s Hospital [25]. Strains of DENV-1 used in the study (and described in Table 1) were passaged once in C6/36 before sequencing. A DENV-1 infectious clone (I.C) was engineered and kindly provided by Dr. Wen Lu (The Army Malaria Institute, Australia). The study was approved by the Queensland University of Technology Research Ethics Unit (Ethics No. 0700000910). As no patient tissue was employed in this study, the University Ethics Unit did not require informed patient consent. All patient identifiers were removed from the dengue virus samples before their use in the research.

**Table 1:** Strains of DENV-1 used.

Strain	Country	Date of Isolation	Accession number	Source	Roche/454 pyrosequencing accession number^a^	Source	Passage number
31459	Myanmar	1998	AY588272	[25]	ERS901694	This study	P1 in C6/36
31987	Myanmar	1998	AY588273	[25]	ERS901695	This study	P1 in C6/36
32514	Myanmar	1998	AY600860	[25]	ERS901696	This study	P1 in C6/36
36957	Myanmar	2000	AY620951	[25]	ERS901697	This study	P1 in C6/36
43826	Myanmar	2001	DQ264966	[15]	ERS901698	This study	P1 in C6/36
44988	Myanmar	2002	AY726552	[24]	ERS901699	This study	P1 in C6/36
47317	Myanmar	2002	KF559253	[24]	ERS901700	This study	P1 in C6/36
47662	Myanmar	2002	DQ265041	[15]	ERS901701	This study	P1 in C6/36
49440	Myanmar	2002	DQ265137	[15]	ERS901702	This study	P1 in C6/36
62690	Myanmar	2005	KF559255	[24]	ERS901703	This study	P1 in C6/36
68417	Myanmar	2007	KF559256	[24]	ERS901704	This study	P1 in C6/36
80579	Myanmar	2009	KF559257	[24]	ERS901705	This study	P1 in C6/36
49440 I.C	Myanmar	2002	KF559254	[24]	ERS901706	This study	1 X BHK, 1 X C6/36

I.C: Infectious clone

^a^: The European Nucleotide Archive

### RNA extraction, RT-PCR and Sanger sequencing

Dengue virus RNA was extracted from 140 μl samples of virus using the QIAamp Viral RNA mini kit (Qiagen), according to the manufacturer’s instructions. RNA was quantified by spectrophotometer. Complementary DNA (cDNA) was produced from the RNA of DENV-1, using random hexanucleotide primers (Boehringer Mannheim) and Expand reverse transcriptase (Expand RT; Roche). Briefly, 1 μl random hexamer primers (200ng/μl) were added to 11 μl RNA in a 0.5 ml tube, and the mixture was incubated at 65°C for 5 minutes in a heating block, before being placed on ice for 2 minutes. Four microliters of 5x RT buffer (Roche), 1 μl 100mM DTT (Roche), 1 μl 10mM dNTPs (Roche), 40 units RNAse inhibitor (Roche), and 50 units 1 μl Expand RT were added to the tube and the volume made up to 20 μl with nuclease free water. RT reactions were incubated at 55°C for 1.5 hours. The primers used for PCR amplification corresponded to a region of the E of DENV-1. These were: D1 843F, 5^/^ATGCCATAGGAACATCC 3^/^ and D1 2465R, 5^/^TTGGTGACAAAAATGCC 3^/^. Five microliters of 10x Expand high fidelity PCR buffer with 15mM MgCl2 (Roche), 1 μl 10mM dNTP, 2 μl forward primer (0.1μmol), 2 μl reverse primer (0.1μmol), 3 units Expand high fidelity enzyme mix (Roche), 5 μl cDNA, and 34.25 μl nuclease free water were mixed to make the total volume of 50 μl. PCR was performed using cycling conditions of 94°C for 2 minutes for one cycle followed by 92°C for 30 seconds, 58°C for 40 seconds and 68°C for 2.30 minutes for 10 cycles, 92°C for 30 seconds, 58°C for 30 seconds and 68°C for 3 minutes for 10 cycles, 92°C for 30 seconds, 58°C for 30 seconds and 68°C for 3.30 minutes for 18 cycles run for 39 cycles followed by 68^°^C for 10 minutes for final extension. PCR products were electrophoresed on 1.0% agarose in 1x TBE buffer, and products of the correct size were gel purified with the MinElute PCR purification kit (Qiagen), according to the manufacturer’s instructions. The purified DNA (100 ng per 300 bp of product) was added to 3.2 pmol of oligonucleotide primers (forward and reverse) in a final volume of 12 μL. The remaining sequencing reaction was performed by the Australian Genome Research Facility Ltd, Brisbane.

### RT- PCR for NGS

Equal amounts of RNA were used for RT. cDNA was produced from the RNA of DENV-1, as in the protocol described above. The primers were designed and used for PCR amplification corresponding to a region of the prM-E-NS1 of DENV-1. These were: D1-330F, 5^/^-AAGTGCTACGGGGTTTCAAG 3^/^ (nt 330–350) and D1-3462R, 5^/^-GACCCTGCAGAGACCATTG 3^/^ (nt 3462–3480). Five microliters of 10x Expand high fidelity PCR buffer with 15mM MgCl_2_ (Roche), 1 μl 10mM dNTP, 2 μl forward primer (0.1μmol), 2 μl reverse primer (0.1μmol), 4 units Expand high fidelity enzyme mix (Roche), 6 μl cDNA, and 32.5 μl nuclease free water were mixed in a 0.6 ml tube to make the total volume of 50 μl. PCR cycling conditions were adopted to obtain maximum DNA products. These, was performed using 94°C for 2 minutes for one cycle and then 94°C for 30 seconds, 58°C for 45 seconds and 68°C for 3.50 minutes run up to 40 cycles and 68°C for 10 minutes for final extension. PCR was performed from the same reaction for all DENVs. PCR products were gel purified with the MinElute PCR purification kit (Qiagen), according to the manufacturer’s instructions. The purified DNA (~1 μg per sample) in a final volume of 10 μL was sent for Roche/454 pyrosequencing. The remaining sequencing reaction was performed at Murdoch University, Perth, Australia.

### Roche/454 FLX Sequencing

Genome fragments of individual strains were amplified from the same PCR reaction from equimolar amounts of RNA and submitted for library preparation before subsequent next-generation sequencing (NGS) using 454 Roche FLX Titanium. Briefly, amplicons were sheared using nebulization to average the fragment size to 800bps. The samples were then taken through library preparations using the rapid library multiplex identifier adaptors in order to enable sample identification following sequencing on the FLX genome sequencer. Samples were then taken through emulsion PCR and run on 1/8th region of a FLX titanium chemistry sequencing run.

### Roche/454 FLX Data Analysis

First, the Roche/454 sequenced reads (study accession number: PRJEB11280) were assessed for any sequencing issues by performing quality checks on the raw sequencing reads obtained with the quality control software, FastQC [26]. A full length phylogenetically relevant consensus sequence (M37726; AY726549, unpublished) generated from this laboratory was used as a reference sequence. The sequenced reads were then mapped to the DENV-1 reference genome M37726 (entire length of 11kbp), using the Burrows-Wheeler Aligner’s Smith Waterman Alignment (BWA-SW) with default parameters [27].

Sequences were also aligned with a nucleotide identity threshold of 95% against the consensus reference sequence with the alignment tool MOSAIK (http://bioinformatics.bc.edu/marthlab/Mosaik), using the recommended parameters for the 454 data to ensure that there was no bias due to the mapping program used. Reads that were not mapped to the reference assembly were then removed from subsequent analysis. This resulted in 1.15% to 10.5% of the raw sequencing reads being removed, and the range of the mean sequencing depth obtained for the PCR-amplified amplicon region being 122-fold to 1,621-fold per residue across the 13 DENV-1 strains.

To identify variants—particularly single-nucleotide polymorphisms (SNPs)—in each DENV-1 population against the reference M37726 genome assembly, LoFreq [28], which is designed to call rare variants from deep sequencing data, was used. In the reference mode, LoFreq reports the variants identified against the reference assembly used, while the consensus mode (-c option) reports variants occurring in the viral population that differs from the consensus genome constructed from the sequence dataset. Therefore, both modes of LoFreq were used in the analysis, with the default parameters applied. Variants were also identified in each virus populations, using VPhaser [29] to compare with the variants identified by LoFreq. To determine the inferred haplotypes and estimate the haplotype frequencies, QuasiRecomb version 1.2 [30] was used to perform a local haplotype reconstruction across the E gene, applying the conservative method, with no recombination assumed and gaps ignored. Since the PCR amplicon spans the prM-E-NS1 gene region, the haplotype inference for the E gene across all 13 samples only considered all sequenced reads that are mapped overlapping the nucleotide positions 935 to 2,419 of the reference M37726 genome assembly.

### Sequence Alignments and Phylogenetic Analysis

Alignment of the Sanger consensus sequences and haplotype sequences derived from 454 reads was performed using the ClustalW program of the Geneious Pro 6.1 software. The aligned nucleic acid sequences were used to construct a bootstrapping phylogenetic tree, using the Neighbor-joining tree building method and Tamura-Nei genetic distance model of the Geneious Pro 6.1.

### Highlighter Plots

Highlighter plots were used to highlight mismatches between nucleotide sequence haplotypes derived from 454 sequence reads. Highlighter plots were performed by using the Gnuplot program in the Los Alamos National Laboratory website-HIV Sequence Database, http://www.hiv.lanl.gov/content/sequence/HIGHLIGHT/highlighter.html.

### Statistical Analysis

Statistical analysis was performed using the GraphPad Prism software package (GraphPad Software Inc.). Unpaired t-test and Analysis of variance (one way ANOVA) were performed to determine the difference between variables. P values lower than 0.05 were considered statistically significant.

## Results

### Phylogenetic analysis

PrM-E-NS1 genes from a total of thirteen DENV-1 strains isolated from 1998 to 2008, including one DENV-1 Infectious Clone (I.C) that was used as a control, were sequenced by Roche/454 pyrosequencing. The E gene consensus sequences of each strain were also derived from Sanger sequencing. The accession numbers for each strain are shown in Table 1. A phylogenetic tree constructed with the consensus DENV-1 E gene nucleotide sequences obtained from Sanger sequencing, showed five distinct branches [24]. DENV-1 complete E gene haplotypes and haplotype frequencies, detected through Roche/454 pyrosequencing, were reconstructed. Reconstructed haplotypes inferred from the viral populations and their frequencies determined through NGS have been used previously to study populations within viruses [30–37].

**Fig 1:**
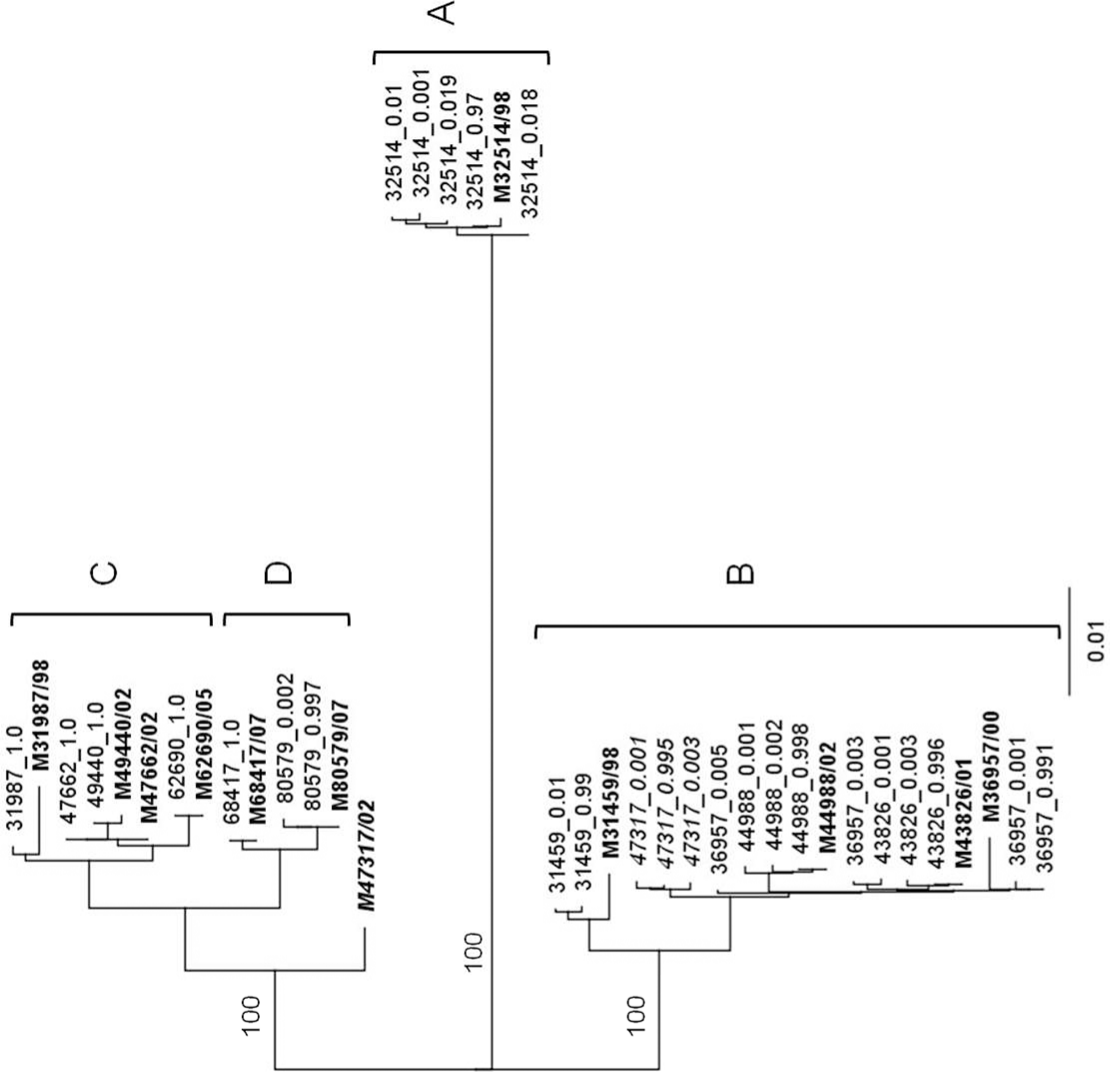
Phylogenetic analysis of the E gene haplotype and consensus sequences of DENV-1. Consensus sequences for each strain have been highlighted. Bootstrap values (100 replications) for key nodes are shown. A distance bar is shown below the tree. Lineage A became extinct in 1998 about the same time lineages B and C appeared. No examples of lineage B have been recovered since 2002, but lineage C still circulated in 2008. Lineage D, first detected in 2006, also still circulated in 2008. For the purposes of analysis, lineages A and B are considered to have become extinct in 1998 and 2002, respectively. Lineages C and D were deemed to be still circulating in 2008.

**Fig 2:**
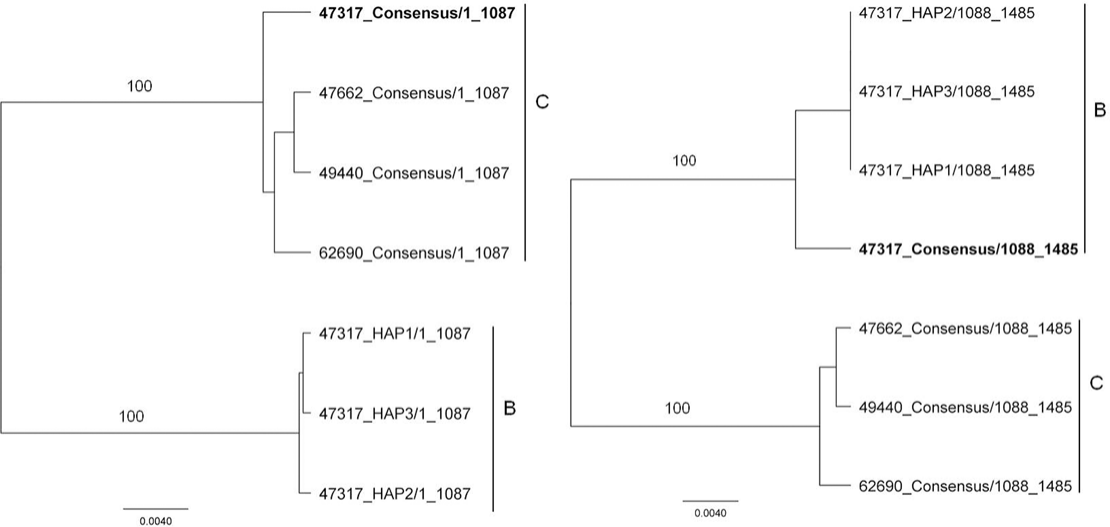
Phylogenetic analyses of nt 1–1087 and nt 1088–1485 of the consensus sequences of the E genes of DENV-1 from lineages B and C and of the sequences of the same regions of the E genes of M47317 haplotypes. Bootstrap values (100 replicates) for key nodes are shown.

A phylogenetic tree was constructed with the consensus DENV-1 E gene nucleotide sequences obtained from Sanger sequencing and the sequences of each constructed haplotype obtained through Roche/454 pyrosequencing, to determine any disparities (Fig 1). With the exception of the M47317 strain, each haplotype segregated with the Sanger consensus sequence of the population from which it was drawn (Fig 1). The intermediate position of the M47317consensus sequence in this tree suggests that it might have been derived from a recombinant genome (Fig 2). Alignment of the consensus nucleotide sequences of representative strains from lineage B and C with the consensus sequence for M47317 and the sequences of M47317 haplotypes, suggested a recombination break point at about nucleotide 1087. Phylogenetic trees constructed with sequences from either side of this point supported this conclusion [i.e., nt 1–1087 of the consensus sequence of M47317 segregated with those from lineage C viruses (Fig 2), while sequences 3^/^ to this point segregated more closely with lineage B viruses and the M47317 haplotypes]. There was no recombination observed within the sequences of M47317 haplotypes with strains from lineage A or D.

### Nature and extent of intra-host genetic diversity in the envelope gene

A combined total of 111,101 raw sequences were acquired from the extinct and skin circulating strains. After quality filtering, an average of 97% of the sequence reads were mapped to the reference (98% were from extinct, and 96% from circulating lineages), using BWA-SW (S1 Table). To ensure there was no bias due to the mapping program used, we also aligned the sequencing reads using MOSIK. There was no significant difference (*p*>0.05, t test) between the two different programs used (S1 Table) in the mean coverage and the percentage of mapping.

Variants in each DENV-1 strain were identified using LoFreq against reference genome. The number of nucleotide changes and the value of these changes are shown in Table 2. Values for strain M47317 were excluded from analysis because of the disparity between the positions in the phylogenetic tree of the haplotypes and the Sanger consensus sequences. DENV-1 I.C was used as control for this analysis. The total variants and variable sites in DENV-1 populations determined by Roche/454 pyrosequencing were greater than DENV-1 I.C. The nucleotide sequence diversity within viral populations was very low, and estimates of the average diversity ranged from 0.009–0.01. There was a significant difference (*p*< 0.03, t-test) in variants identified between extinct (A, B) and extant (C, D) lineages, however, no difference (*p*> 0.05, t-test) for any other parameters measured in Table 2. While 1.15% to 10.5% of the sequencing reads could not be assembled, there was no significant difference (*p*>0.16, t-test) observed in extinct and circulating lineages (S1 Table).

**Table 2:** Measures of intra-host genetic diversity among DENV-1 lineages. Genetic diversity was measured through SNPs analysis.

DENV-1 lineage	Name of the lineage	Strain	Total reads analysed	Mean Coverage/nt	Total nt variants ^a^	Total number of variable sites		Est. mean genetic diversity (%)^*c*^	
						n.t	a.a	n.t	a.a
Extinct	A	M32514	5919	454.20	130	10	0	0.023	n.a
	B	M31459	8330	736.47	51	0	0	n.a	n.a
		M36957	12127	1091.10	57	1	0	0.011	n.a
		M43826	3294	273.73	57	0	0	n.a	n.a
		M44988	15211	1334.72	59	0	0	n.a	n.a
Survived	C	M31987	17729	1621.37	5	0	0	n.a	n.a
		*M47317* ^*b*^	9333	824.04	*56*	*0*	*0*	*n*.*a*	*n*.*a*
		M47662	6300	561.02	4	1	0	0.009	n.a
		M49440	13439	1217.48	5	0	0	n.a	n.a
		M62690	3126	287.08	7	1	0	0.002	n.a
	D	M68417	8635	771.78	19	0	0	n.a	n.a
		M80579	1384	122.77	22	1	0	0.010	n.a
Control		I.C	6274	697.80	15	0	0	n.a	n.a

n.a; not applicable

^a^ The number of bases that are different from the reference genome.

^*b*^ Genetic diversity of recombinant DENV-1 strain showing in italic.

^*c*^ Estimated mean genetic diversity $=\frac{TotalVariants}{E\left(Depth\right)\;x\;Length}\;=\frac{\sum_{i=0}^{n}{FreqVariant}_{i}\;x\;{Depth}_{i}}{\frac{\sum_{i=0}^{n}{Depth}_{i}}{n}\;x\;Length}$ where *n* is the total number of variants.

We identified variable sites in the viral community of both lineages; however, there was no trend observed (Table 3). Recent studies showed that the defective viruses that contained intragenic stop codon mutations, which are most likely to be deleterious, can be transmitted in nature through complementation with functional virus [15]. While we hypothesised that an accumulation of deleterious mutations over time led to the extinction and replacement of DENV lineages, there were no stop codon, frameshift and non-frameshift mutations observed in the E genes of any lineage in this study. Nor were there any insertions or deletions observed in the E genes of any lineage.

**Table 3:** Detecting variants, stop codons and deletions using two different sequence analysis platforms.

Lineage Status	Strain	Year	Detected by LoFreq				Detected by VPhaser			
			Total Variants ^a^	Stop Codons	Frameshift	Non-Frameshift	Total Variants ^a^	Stop Codons	Frameshift	Non-Frameshift
Extinct	32514	1998	130	0	0	0	148	0	4	0
	31459	1998	51	0	0	0	60	1	7	1
	36957	2000	56	0	0	0	62	1	9	2
	43826	2001	57	0	0	0	62	0	10	0
	44988	2002	59	0	0	0	68	1	2	0
Circulating	31987	1998	5	0	0	0	12	0	10	1
	*47317* ^*b*^	*2002*	*56*	*0*	*0*	*0*	*58*	*1*	*7*	*1*
	47662	2002	3	0	0	0	4	0	0	0
	49440	2002	5	0	0	0	21	0	7	2
	62690	1995	6	0	0	0	12	0	1	0
	68417	2007	19	0	0	0	74	0	13	0
	80579	2008	21	0	0	0	22	0	0	0
Control	I. C	-	15	0	0	0	15	0	0	0

^a^ The number of bases that are different from the reference genome.

^*b*^ Genetic diversity of recombinant DENV-1 strain showing in italic.

To identify any bias in the results due to the analysis platform used, we also identified variants using VPhaser. There was a significant difference (*p*<0.001, one way ANOVA) in the results observed between two platform used (Table 3). Few strains in both lineages presented a stop codon; however, this was a result of a homopolymer associated with an alignment error at position 1046 A->T, K38. Although frameshift and non-frameshift mutations were detected in both lineages using VPhaser, there was no significant difference (*p*>0.05, t-test) identified between the two lineages (Table 3).

**Fig 3:**
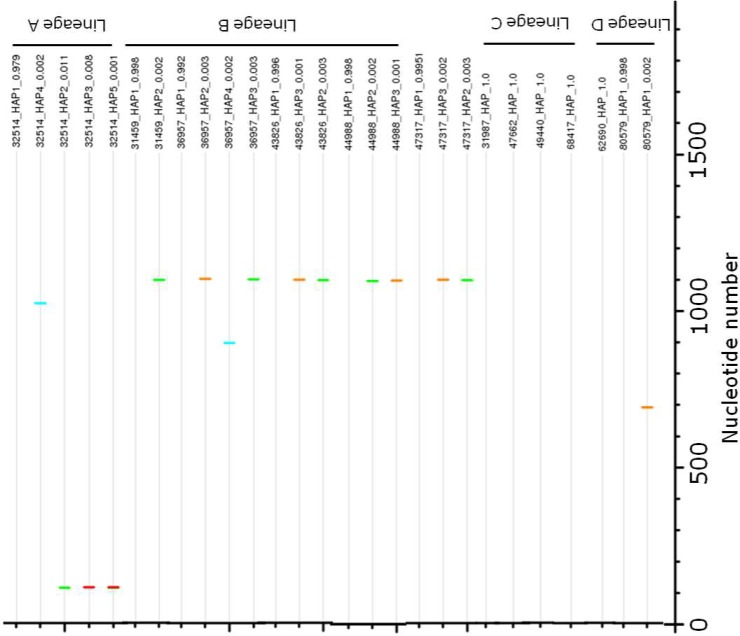
Distribution of polymorphic sites in the E genes of haplotypes of DENV-1 Lineage A, B, C and D. Changes from the most prevalent haplotypes/consensus are shown as A: green, T: red, G: orange, C: light blue. The percentage of each haplotype in the population is shown in parenthesis.

The distribution and the nature of the changes (relative to the sequence of the most common haplotype/consensus) within the E gene are shown in Fig 3. Few strains in either lineage displayed polymorphisms in their complete E genes. Overall, only 1 to 5 variable sites were observed. No polymorphic positions between extinct and extant lineages were conserved, and there were no conserved substitutions (e.g., A or T or G or C) that were exclusive to either extinct or extant lineages. The most polymorphic sites were observed between nucleotides 800 to 1100 in an extinct lineage; however, the frequency of these polymorphisms between extinct and extant lineages was not significantly different (*p*>0.69, t test). Studies have shown that the DENV E domain (D) II contains the highest diversity of all viral genome domains [21]. However, in this study, no domain (DI-DIII) bias in conservative and non-conservative changes between extinct and extant lineages was observed.

## Discussion

RNA virus populations must strike a fine balance between the genetic diversity necessary to survive in changing host environments [9] and the genetic diversity leading to error catastrophe. Descriptions of the nature and magnitude of genetic diversity in DENV populations [15, 17, 38–40] have suffered from two technological constraints: the limited size of the genome sequence that can be cloned into a single plasmid for subsequent sequencing, and the logistics of sequencing statistically large numbers of individually cloned genome fragments.

We investigated intra-host genetic diversity of DENV-1 using NGS technology as it has the potential to address both issues. As this technology has recently become routinely available in the field, a study to determine whether its use is appropriate for the determination of intra-host genetic diversity is timely and essential. To explore this question, we analysed DENV-1 E gene sequences from each of the populations chosen for this study. The DENV-1I.C clone E gene was chosen as the unit of analysis by NGS, and was also Sanger sequenced to identify potential sequencing errors and artefacts that might be inherent in the NGS approach.

Our data are largely consistent with those reported in the literature. The genetic diversity in DENV-1 populations determined by 454 pyrosequencing was greater than control I.C, but was less than the previous estimates determined by conventional cloning and sequencing [17, 40, 41]. This observation is consistent with a recent study [20, 21] showing that the nature and magnitude of intra-host genetic diversity in DENV-2 determined by 454 sequencing was significantly lower than previous estimates determined by cloning and sequencing [17, 40, 41].

There was no correlation observed between the nature and extent of the genetic diversity of the DENV-1 E genes and the observed extinction or survival of the lineage from which they were drawn (*p*>0.5). There were no stop codon, frameshift and non-frameshift mutations observed in the E genes of any lineage. There were no lineage-specific nucleotide substitutions or changes in the DENV E gene that were likely to have contributed to lineage extinction, nor were there substitutions or changes identified that were likely to have provided a fitness advantage to the circulating lineages.

Recently, the DENV E domain (D) II was shown to contain the highest diversity of all viral genome domains [21]. Because E (D) II is highly immunogenic in humans [42], the higher genetic diversity in E DII was possibly due to immune-driven pressure. However, no significant differences in genetic diversity in envelope protein domains that were likely to cause lineage extinction were detected. This is consistent with previous studies [43, 44] that were also unable to detect specific changes in the DENV genomes that might be responsible for lineage extinction. Although no conserved changes were observed in extinct lineages relative to circulating lineages, a few polymorphic sites were identified in all populations of lineage B, particularly at E1083 and E1085. These are synonymous mutations, and do not have any effect on the function of the protein that can result in lineage extinction. However, the nature and magnitude of the genetic diversity of the E genes, determined by 454 sequencing of the DENV-1 in this study (Table 2), was 10- to 100- fold less than previously published reports of DENV diversity [15, 17, 40, 41], and this might have affected the subsequent analysis.

This observation of the nature and extent of intra-host genetic diversity in DENV-1 is consistent with recent reports of the diversity determined by 454 pyrosequencing [20, 21] and by cloning and Sanger sequencing [40], but significantly lower than some previous estimates determined by cloning and Sanger sequencing [15, 17]. Twenty clones from strain M47317 contained 78 polymorphic sites and 1 stop codon, and 20 clones of M49440 contained 89 polymorphic sites and 11 stop codons [15]. The same strains analysed by NGS contained 4 (M47317) and 2 (M47440) polymorphic sites and no stop codons. However, these comparisons have been employing from cDNA generated from different stocks of virus using different primers.

There is a need for comparisons of diversity determined by NGS and by cloning and sequencing of the same cDNA stocks. There is also the need for all NGS sequencing reads to be included in analyses of SNPs rather than only those that can be assembled into complete E gene sequences. However, we acknowledge that an appropriate comparison between pyrosequencing and cloning and Sanger might not be possible due to the amount of the genome that can be cloned, and the difference in platforms and parameters/filters used for sequence analysis. The NGS FLX (454 Life Science, Roche) technology was chosen for this study because it provides longer sequence lengths (average of 300 bases) than other platforms, demonstrates 99.5% accuracy in single-read, and exhibits a very low incidence of substitution errors (<10–6) [45, 46]. Because of the algorithms used to assemble the short reads generated from 454 sequencing and reconstruct haplotypes of the assembled sequences, the possibility of excluding genuine diversity must be considered. Less than 1.15% to 10.5% of sequence reads could not be assembled with the algorithms employed. Therefore, some diversity could be excluded by the 10% (upper estimate) of sequences that left out of contigs because they are ‘error’. However, there was no trend observed in the proportion of sequences that were excluded in extinct and extant lineages (S1 Table).

As reported in Romano’s study [20], SNPs (A to G) that are detected in the standard cloning and Sanger sequencing also appeared in 454 sequencing reads (at frequencies about 0.4%), but in homopolymer regions of the 454 sequencing reads. Therefore, the SNPs were excluded from the deep sequencing analysis. This was due to the thresholds/filters adopted in the analysis to consider a variant to be true (1% frequency and quality score above 30). However, genetic diversity that excluded when algorithm removes bases with low quality scores [e.g. hypermutated sequence (sequences with more than 5 nt mutations within the first 20 positions of the read in either of the ends); reads with indels that resulted in a frame-shift or variants with higher allele frequencies (5%)] is likely the sequencing errors. Additionally, the algorithms might consider singletons (a class of polymorphism) as errors, and subsequently “correct” the errors, despite the fact that some mutations might represent true biological variants [40].

In this study, variants that identified in each virus populations using the VPhaser analysis platform were compared with the variants identified by the LoFreq platform. There was a significant difference (*p*<0.001) in the results observed between two platform used (Table 3). We did not identify any stop codon mutation when using the LoFreq platform analysis. While a stop codon mutation appeared in both lineages DENV-1 populations when analysed with VPhaser (Table 3), this mutation appeared in a homopolymer region and was unlikely to represent a true biological variant. While there was no deletion observed when analysed using LoFreq platform, deletions were detected when analysed using VPhaser. This highlighted that there was some discrepancy between sequence diversity estimated by NGS analysis platforms, and indicates the need to evaluate these platforms in order to comprehensively determine intra-host viral genetic diversity.

To our knowledge, this is the first study to investigate the intra-host genetic diversity in DENV-1 E gene by NGS technology. The DENV E gene was chosen based on the considerable body of evidence suggesting that the nature and magnitude of the diversity in DENV E genes is similar to that in most other regions of the open reading frame, and is therefore representative of genomic diversity [21, 39, 43]. Most data relating to diversity in DENV has thus far been obtained from the E gene. This allows for a more comprehensive comparison of our data with the data in the existing literature.

DENV frequently experiences intra- and inter-host population bottlenecks during transmission. The substructure of DENV populations could substantially impact the effects of bottleneck transmission due to the phenotypic variability within the population [24]. We hypothesised that the accumulation of deleterious mutations over time led to the extinction and replacement of DENV lineages. Although this hypothesis was ultimately not supported by our data, the data does provide insights into the DENV population substructures, and extends our understanding of their complexity. Finally, our experimental design revealed a glaring discrepancy between sequence diversity estimated by NGS analyses and the diversity estimated by the classic method of cloning and Sanger sequencing. For NGS to be considered reliable for diversity analyses, this discrepancy must be reconciled.

## Supporting Information

S1 Table Mapping 454 pyrosequencing reads using two different sequence analysis programs. (DOCX)
