# Supplementary material for: Nature and Extent of Genetic Diversity of Dengue Viruses Determined by 454 Pyrosequencing
Source: PLoS One. 2015 Nov 13;10(11):e0142473. doi: 10.1371/journal.pone.0142473 (PMC4643897; doi:10.1371/journal.pone.0142473)
Supplement: S1 Table — (DOCX) [file pone.0142473.s001.docx]

**S1 Table. Mapping 454 pyrosequencing reads using two different sequence analysis programs.**

| **Strain** | **Lineage Status** | **% GC** | **BWA-SW alignment** | | |  | **MOSAIK alignment** | | |  |
| --- | --- | --- | --- | --- | --- | --- | --- | --- | --- | --- |
|  |  |  | **Mean Coverage** | **% Covered** | **% Mapped** | **Total** | **Mean Coverage** | **% Covered** | **% Mapped** | **Total** |
| 32514 | Extinct | 47 | 454.2 | 100 | 95.12 | 5919 | 502.23 | 100 | 98.25 | 5895 |
| 31459 | Extinct | 45 | 736.47 | 100 | 98.13 | 8330 | 787.65 | 100 | 98.87 | 8247 |
| 36957 | Extinct | 45 | 1091.1 | 100 | 98.02 | 12127 | 1169.21 | 100 | 98.94 | 12021 |
| 43826 | Extinct | 46 | 273.73 | 100 | 89.5 | 3294 | 293.48 | 100 | 90.48 | 3268 |
| 44988 | Extinct | 45 | 1334.72 | 100 | 97.73 | 15211 | 1437.61 | 100 | 98.82 | 15118 |
| 31987 | Circulating | 46 | 1621.37 | 100 | 97.61 | 17729 | 1694.81 | 100 | 97.41 | 17458 |
| 47317 | Circulating | 45 | 824.04 | 100 | 98.02 | 9333 | 887.71 | 100 | 99 | 9267 |
| 47662 | Circulating | 46 | 561.02 | 100 | 96.21 | 6300 | 591.73 | 100 | 96.96 | 6275 |
| 49440 | Circulating | 45 | 1217.48 | 100 | 98.68 | 13439 | 1283.47 | 100 | 99.33 | 13375 |
| 62690 | Circulating | 45 | 287.08 | 100 | 98.62 | 3126 | 300.06 | 100 | 98.84 | 3097 |
| 68417 | Circulating | 45 | 771.78 | 100 | 98.53 | 8635 | 817.14 | 100 | 99.27 | 8587 |
| 80579 | Circulating | 45 | 122.77 | 100 | 97.18 | 1384 | 128.99 | 100 | 97.51 | 1368 |
| Infectious Clone | Control | 45 | 697.8 | 100 | 98.85 | 6274 | 732.69 | 100 | 99.57 | 6245 |
